# An Infant Milk Formula Supplemented with Heat-Treated Probiotic *Bifidobacterium animalis* subsp. *lactis* CECT 8145, Reduces Fat Deposition in *C. elegans* and Augments Acetate and Lactate in a Fermented Infant Slurry

**DOI:** 10.3390/foods9050652

**Published:** 2020-05-19

**Authors:** Ángela Silva, Nuria Gonzalez, Ana Terrén, Antonio García, Juan Francisco Martinez-Blanch, Vanessa Illescas, Javier Morales, Marcos Maroto, Salvador Genovés, Daniel Ramón, Patricia Martorell, Empar Chenoll

**Affiliations:** 1 Health & Wellness-ADM Nutrition-ADM Biopolis, 46980 Paterna, Spain; angela.silva@adm.com (Á.S.); nuria.gonzalez@adm.com (N.G.); Salvador.genoves@adm.com (S.G.); daniel.ramonvidal@adm.com (D.R.); patricia.martorell@adm.com (P.M.); 2 Instituto Fundación Teófilo Hernando, 28029 Madrid, Spain; ana.terren@ifth.es (A.T.); agg@uam.es (A.G.); marcos.maroto@ifth.es (M.M.); 3 Departamento de Farmacología y Terapéutica, Universidad Autónoma de Madrid, 28029 Madrid, Spain; 4 Instituto de Investigación Sanitaria, Hospital Universitario de La Princesa, Facultad de Medicina, 28006 Madrid, Spain; 5 Health & Wellness-ADM Nutrition-ADM LIfesequencing, 46980 Paterna, Spain; juan.martinezblanch@adm.com (J.F.M.-B.); Vanessa.illescas@adm.com (V.I.); 6 Product Development Department, Alter Farmacia SA, 28880 Madrid, Spain; jmoraleso@grupo-alter.com

**Keywords:** heat-treated probiotic, *Bifidobacterium animalis* subsp. *lactis*, fat deposition, acetate, lactate, *C. elegans*

## Abstract

Pediatric obesity has a growing health and socio-economical impact due to cardiovascular and metabolic complications in adult life. Some recent studies suggest that live or heat-treated probiotics have beneficial effects in preventing fat deposition and obesity in preclinical and clinical sets. Here, we have explored the effects of heat-treated probiotic *Bifidobacterium animalis* subsp. *lactis* CECT 8145 (HT-BPL1), added as a supplement on an infant milk formula (HT-BPL1-IN), on *Caenorhabditis elegans* fat deposition and short-chain fatty acids (SCFAs) and lactate, using fermented baby fecal slurries. We have found that HT-BPL1-IN significantly reduced fat deposition in *C. elegans,* at the time it drastically augmented the generation of some SCFAs, particulary acetate and organic acid lactate. Data suggest that heat-treated BPL1 maintains its functional activities when added to an infant powder milk formula.

## 1. Introduction

A pool analysis of 128.9 million children, adolescents, and adults reveals the huge health impact of obesity [1]. This is illustrated by several studies. For instance, adults with a history of chilhood obesity have higher prevalence of metabolic syndrome and cardiovascular risk [2,3]. Additional complications of pediatric obesity are poor skeletal maturation [4], psychosocial inadaptation [5], or early inflammatory status [6].

During the last years, new strategies derived from the gut microbiome analysis are emerging to understand and combat childhood obesity. Thus, an association of early life gut microbiota with body mass index has been demonstrated in an observational study in 0–3 years age infants [7]. Such association seems to be linked to short-chain fatty acids (SCFA) binding G protein coupled receptor (GPR41), which modulates host adiposity [8]. These findings point to a potential use of prebiotic, probiotic or symbiotic products to modify in some way the gut microbiota in individuals prone to obesity and to reduce the impact of this dysbiosis in childhood and later adulthood. Specific probiotic supplements can impinge on lower incidence of obesity and hypertension [9,10,11,12]. Other studies have also shown that specific probiotics impact on weight management in adults [10,11,12]

Recent studies have shown that heat-treated bacteria release metabolites or cell components that can display functional actions, such as immunomodulation or antagonism of pathogens [13,14,15]. This has led to an increasing interest in using heat-treated probiotics [13,14,15,16]. In fact, a few clinical trials have shown heat-treated bacteria functional activities in for instance infantile colic [17]) or in functional bloating [18].

In a set of preclinical studies, strain *Bifidobacterium animalis* subsp. *lactis* strain BPL1 was isolated from infant feces and deposited in Spanish Type Culture Collection (CECT) under accession number CECT 8145 and, in a massive screening, demonstrated its capacity to reduce fat deposition [19]. Initially, the strain was tested for its ability to reduce fat content in *Caenorhabditis elegans* model [19], and later in two murine models of obesity; one carried out in conventional wistar rats fed on a cafeteria diet [20] and the second based on the use of genetically obese rats Zücker rats [21]. In all three studies, the animals were fed BPL1 from infancy/larvae state to young adulthood, and in all three cases, an effect of lifetime probiotic consumption on adult fat was observed. In the two murine model studies, it was also observed that probiotic intake in lean individuals had no effect on their body fat levels or other measured parameters. Moreover, both in the *C. elegans* model and in the study in rodents on a cafeteria diet, promising results were obtained with a thermally treated version of the probiotic strain, pointing to a non-life-dependent mode of action, as a part of BPL1 effectiveness. However, while these results showed an effect of BPL1 cells on fat deposition in both animal models and clinical human trial, we were unable to study the potential involvement of the gut microbiome in the observed functionality. This potential involvement is of great importance in order to be able to analyze in depth and systematically the potential mechanisms of action of the probiotic strain (by direct action and by the modulating effect of the microbiome). Thus, further to these results, in this study we considered to evaluate whether the effect observed in animals and humans was due to a direct action of the cells, or if there was an impact on the bacterial microbiome and compounds derived from its metabolism that could have an effect on its functionality in the preclinical model of *C. elegans*. Secondly, and in conjunction with the previous point, we defined as an objective to study whether the addition of the probiotic to a commercial product (in this case infant milk formula) could have a positive or negative impact (reinforcing or diminishing functionality respectively) on its effect.

In the present study, a mixed fecal slurry model was used to analyze the impact of HT-BPL1 strain and infant formula containing the heat-treated probiotic on microbiome, and its potential synergistic effect with the infant milk formula. Fecal slurry approach enabled us to ex vivo analyze together with microbiome changes by massive sequencing, SCFAs production and, furthermore, the impact of this microbiome modulation on *C. elegans* fat deposition.

## 2. Materials and Methods

### 2.1. In Vitro Fecal Fermentations

*Bifidobacterium lactis* BPL1 cells were grown anaerobically in de Man, Rogosa, and Sharpe medium (MRS; Oxoid, Basingstoke, UK), supplemented with cysteine (0.05% wt/vol; Sigma, St. Louis, MO, USA; MRS-C) at 37 °C for 18 h. Cells were then harvested by centrifuging and viability evaluated by plate counting in MRS-C agar (anaerobically, 37 °C for 48 h). After that, cells were heat treated (autoclaved at 121 °C for 20 min) and the absence of viability checked by broth culturing in MRS anaerobically at 37 °C for 18 h. Fecal fermentations were carried out by using 3 mL fermentations in 10 mL fermentor (24 multi-well plate; µ-24 Bioreactor, Applikon Biotechnology, Delft, The Netherlands). Fresh fecal samples came from 3 months babies fed with a milk formula free of probiotics. Immediately after being obtained, stools were vacuum stored in refrigerated conditions until the fermentation experiments were performed. Fresh feces were weighted and dissolved in pre-reduced Mc Bain and MacFarlane medium [22] in a ratio 1:5 (feces:medium) to hydrate them. Three milliliters of the homogenized mixture (fecal slurry) were inoculated in each fermentor, and heat-treated BPL1 (HT-BPL1) was added alone (HT-BPL1), or included in the infant formula Innova, from Nutriben, Alter, Madrid, Spain (HT-BPL1-IN). Medium was previously degassed and headspace was removed before starting fermentation with nitrogen. All the protocol was performed in an anaerobic cabinet. All fermentors were maintained under a head space of oxygen-free nitrogen and were continuously stirred. Anaerobiosis, temperature (37 °C) and pH (pH 5.5) were maintained constant along the fermentation period that was run for 24 h. Slurry samples were anaerobically taken and aliquoted for further processing and analysis.

### 2.2. Body Fat Monitoring in C. elegans

Fresh samples were analysed in *C. elegans* model. The effects of fecal slurry samples subjected to different treatments, on body fat content in *C. elegans*, were analysed as previously described [19]. Briefly, concentrated *C. elegans* cultures (50 µL, OD = 30) were added on top of a NGM surface previously seeded with Escherichia coli OP50, to ensure adequate nutrition conditions. Positive controls were NGM plates with anti-obesity drug Orlistat (6 µg/mL).

Lipid content in worms was determined by adding 0.05 µg/mL of Nile red to NGM agar plates containing the different fecal treatments. Worms were incubated for 3 days at 20 °C, until they reach the adult stage. Then, nematode samples placed in M9 buffer were assayed for their fluorescence in a FP-6200 system (JASCO Analytical Instruments, Easton, MD, USA). Excitation wavelength was at 480 nm and emission was at 571 nm. A total of 120 worms per condition were analyzed in each of 2 different experiments.

### 2.3. Organic Acids Analysis

To analyze short chain fatty acids (SFCAs) acetate, propionate, butyrate and succinate, and organic acid lactate at the end of the experiment, a volume of 500 µL of fermentation was centrifuged at 14,000 rpm for 60 min, filtered through a Millipore 0.45 μm pore-size filter (Billerica, MA, USA), and diluted ½ in MilliQ quality water. The quantification was conducted on a HPLC Acquity equipped with an Aminex HPX-87H 300 × 7.8 mm column (BioRad) under conditions defined by manufacturer. Detection was achieved by a refractive index detector. The eluent was H_2_SO_4_ 5 mM with an isocratic flow rate of 0.6 mL/min. No internal standard was included. Quantification was performed by the use of standard curves with correlation coefficient *R*^2^ > 0.999.

### 2.4. Microbiome Analysis by Deep Sequencing

Slurry samples were immediately frozen and stored at −20 °C until their processing. DNA from samples was isolated following Yuang and co-workers [23] with minor modifications to avoid bias in DNA purification toward misrepresentation of gram positive bacteria, with the aid of MagnaPure Compact System (Roche Life Science). For massive sequencing, the hypervariable region V3-V4 of the bacterial 16s rRNA gene was amplified using key-tagged eubacterial primers [24] and sequenced with a MiSeq Illumina Platform, following the Illumina recommendations for Library preparation and sequencing.

The resulting sequences were split, taking into account the barcode introduced during the PCR reaction. Quality control of the sequences was performed in different steps. Firstly, quality filtering (with a minimum threshold of Q20) was performed using fastx tool kit version 0.013, then primer (16s rRNA primers) trimming and length selection (reads over 300 nts) was done with cutadapt version 1.2. (https://cutadapt.readthedocs.io/en/stable) [25]. FASTQ files were converted to FASTA files and UCHIME program version 7.0.1001 [26] was used in order to remove chimeras that may arise during the amplification and sequencing step. Then clean FASTA files were BLAST against NCBI 16s rRNA database using blastn version 2.2.29+. The resulting XML files were processed using a python script developed by Lifesequencing S.L. (Paterna, Valencia, Spain) in order to annotate each sequence at different phylogenetic levels (phylum, family, genus and species). Alpha diversity was conducted using specaccum program in the vegan package, as implemented for R version 3.2.3. Presence results are expressed as the percentage of each group sequences.

### 2.5. Statistical Analysis

Results are given as the mean ± standard deviation. Differences between the groups were analyzed using Tukey’s multiple comparison tests. Data on fat deposition in *C. elegans* were analyzed by Student’s *t* test. Statistical analyses were performed with GraphPad Prism 8.4.1 software (San Diego, CA, USA), setting the level of statistical significance at 5%.

## 3. Results

### 3.1. Effects of a Milk Powder Supplemented with Heat-Treated BPL1, on C. elegans Fat Deposition

**Figure 1:**
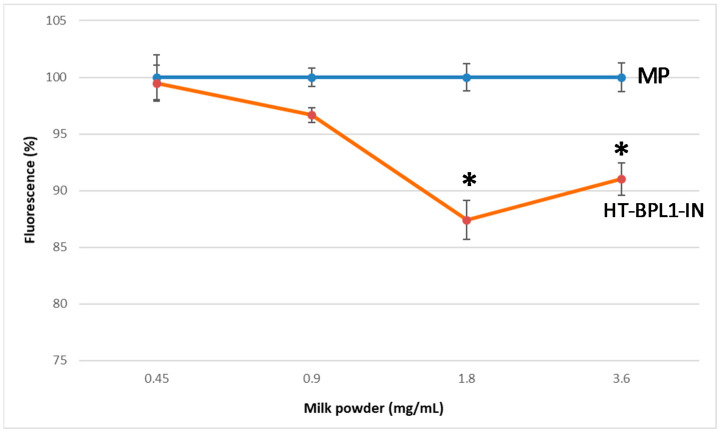
Milk powder supplemented with heat-treated BPL1 (HT-BPL1-IN), reduced fat deposition in *C. elegans* measured as fluorescence with red Nile, in a concentration-dependent manner. Worms were incubated for 3 days with increasing concentrations (abscissa) of milk powder alone (MP) or milk powder supplemented with HT-BPL1-IN. Data are means ± SD of 120 worms assayed in a prototype experiment. * *p* < 0.001 with respect control.

First we explored whether an infant milk powder added alone (MP) or MP supplemented with HT-BPL1 (HT-BPL1-IN, INNOVA), exerted a functional effect on *C. elegans* fat content. Worms were incubated by 3 days with milk powder at increasing concentrations. Figure 1 shows that fat body content (monitored as relative fluorescence of Nile Red, ordinate) was unchanged at the four concentrations of control MP. However, in the case of worms exposed to HT-BPL1-IN, there was a significant 12.5 ± 1.7% reduction (*p* < 0.001) at the concentration of 1.80 mg/mL and 9.0 ± 1.4% reduction at the concentration of 3.60 mg/mL (*p* < 0.001). This preliminary result prompted another set of experiments through the incubation of fermented baby slurries in the presence of HT-BPL1-IN.

**Figure 2:**
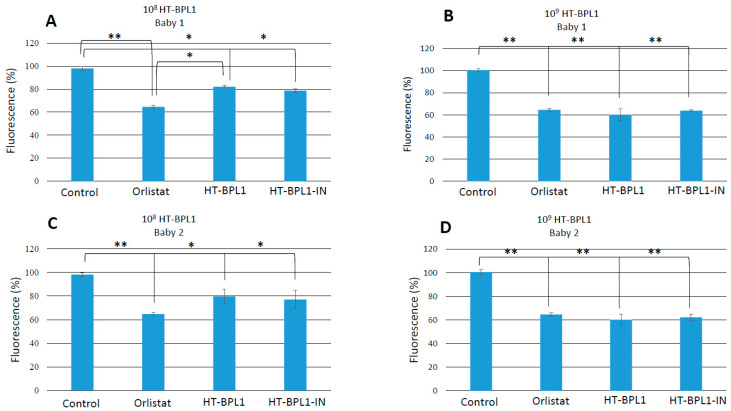
Effects of heat treated BPL1 (HT-BPL1) alone, or added on the infant milk formula Innova (HT-BPL1-IN), on fat deposition of *C. elegans* grown for 3 days on the fermented slurries (24 h) from two different healthy babies. Fat deposition was monitored with relative fluorescence (ordinates) at the end of the incubation periods, expressed as % reduction of fluorescence compare to control worms (worms grown on DMSO). Antiobesity drug Orlistat was added to a well at 6 µg/mL as positive control (DMSO, the solvent for Orlistat). (**A**,**C**) experiments done with 10^8^ HT-BPL1 cells/reactor in slurries from babies 1 (**A**) and 2 (**C**). (**B**,**D**), experiments done with 10^9^ HT-BPL1/reactor with innova milk formula (HT-BPL1-IN) or HT-BPL1 alone, in fermented slurries from baby 1 (**B**) or baby 2 (**D**). Data are mean ± SD of 120 worms per well in each condition * *p* < 0.05, with respect to control. ** *p* < 0.001, with respect to control.

Experiments were done at two concentrations of HT-BPL1, equivalent to 10^8^ and 10^9^ cells per reactor (Figure 2). Worms were grown for 3 days in 24-h fermented fecal slurries from two different infants. The antiobesity drug Orlistat was used as a positive control. In the presence of HT-BPL1, fat reduction accounted for 17.8 ± 1.1% (*p* < 0.05) in baby 1 slurry and 20.2 ± 6.0% (*p* < 0.05) in baby 2. In HT-BPL1-IN assays, reductions in fat deposition with respect to control were 21.3 ± 1.6% (*p* < 0.05) in baby 1 and 22.8 ± 7.9% in baby 2 (*p* < 0.05). In spite of those pronounced reductions, they did not reach the level of statistical significance compared to HT-BPL1.

The reduction in fat deposition observed in worms grown in the presence of 10^9^ heat-treated bacteria was more notable. In all cases, HT-BPL1 reduction was close to Orlistat results. HT-BPL-1 reduced fat deposition by 39.8 ± 5.1% (*p* < 0.001) and 36.2 ± 1.0% in the presence of HT-BPL1-IN (*p* < 0.001). In baby 2 slurry, decreased fat deposition was 39.8 ± 4.6% for HT-BPL1, and 37.8 ± 2.7% for HT-BPL1-IN (*p* < 0.001 for the two treatments).

### 3.2. Effects of HT-BPL1, Alone or Included in an Infant Milk Formula, on the Production of Short-Chain Organic Fatty Acids

This study was aimed at finding out how HT-BPL1 could affect the level of SCFAs and organic acids generation in fecal slurries from healthy infants, before (0 h) and after fermentation for 24 h. 

**Figure 3:**
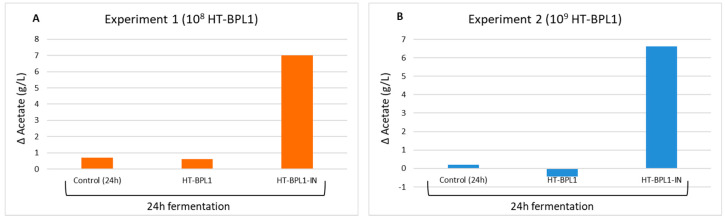
Net increase of acetate in fecal baby slurries fermented for 24 h, in the absence (control) and the presence of HT-BPL1 alone or HT-BPL1 included in an infant milk formula (Innova) supplemented with HT-BPL1 cells (HT-BPL1-IN). (**A**) experiment 1 done with a baby slurry in the presence of 10^8^ HT-BPL1 cells; (**B**), experiment done with a baby slurry in the presence of 10^9^ HT-BPL1 cells. Data calculated from Table 1, by subtracting the background level of acetate in a non-fermented slurry (0 h in the table).

Table 1 summarizes the results obtained. Figure S1 shows representative chromatograms obtained in assays 1 and 2. Note that neither propionic acid nor butyric acid underwent significant changes with respect to control (0 h), both in assay 1 and assay 2. Neither, slurries incubated with HT-BPL1 alone for 24 h, experimented any change with respect to control, both in the case of acetic acid or lactic acid. However, when fermentation was done in the presence of HT-BPL1-IN, acetic acid augmented over 3-fold compared to 0 h samples (9.89 ± 0.02; *p* < 0.001). The same occurred with lactic acid that rose from near undetectable levels at 0 h (0.08 ± 0.11 g/L) to 8.34 ± 0.20 g/L (*p* < 0.001). In assay 2 these differences were maintained even though in this assay acetate and lactate baseline levels were higher in the control samples (Table 1). In Figure 3, the net increases of acetate in fermented fecal slurries, are plotted.

### 3.3. Effects of a Milk Powder Supplemented with Heat-Treated BPL1, on Microbiome

Microorganisms present in fecal slurries were identified and quantified in terms of relative abundance. Diversity by Shannon Index at genus level was evaluated (Supplementary Figure S2), identifying some differences between babies after 24 h of fermentation in some of the trials, and showing a later homogeneity once BPL1-HT or BPL1-HT-IN is added, with slight differences between them.

**Figure 4:**
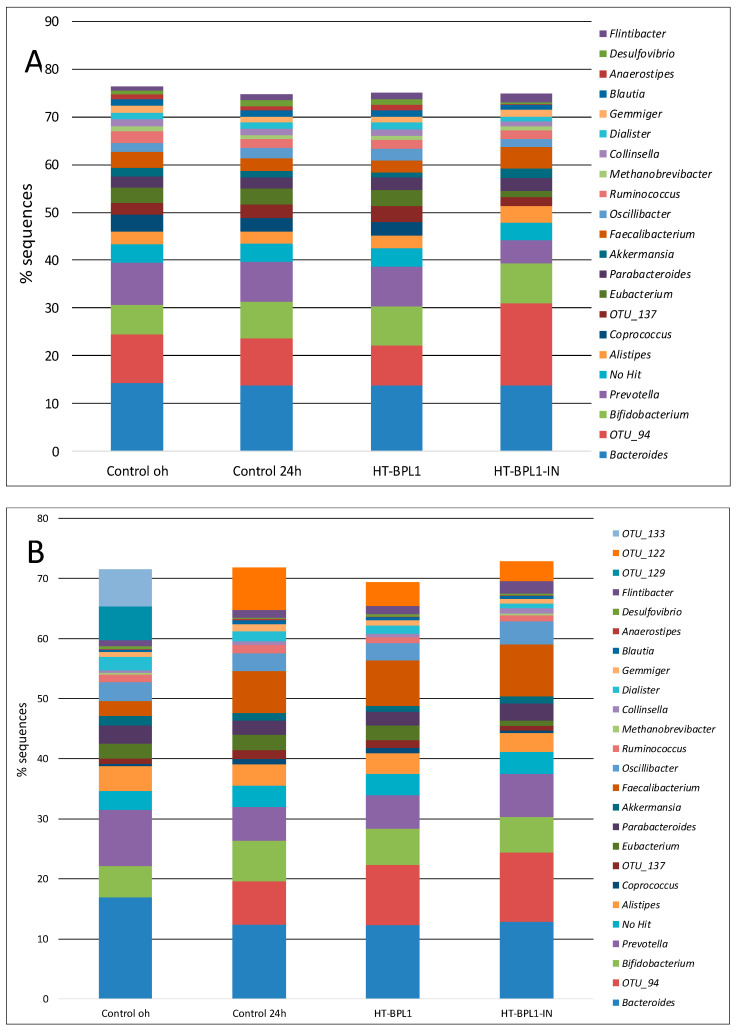
Percentage of identified microorganisms by 16s rRNA by Illumina V3-V4 sequencing. (**A**) experiments run with two different baby slurries in the presence of 10^8^ HT-BPL1 cells; (**B**) experiments run with two different baby slurries in the presence of 10^9^ HT-BPL1 cells.

When we analyzed the potential effect on the regulation of microbiome at genus level in assays with 10^8^ cells HT-BPL1 incorporated (Figure 4A), we observed that fermentation decreased the presence of genera Bacteroides and *Prevotella*, both in majority presence, and increased the presence of the genus *Fecalibacterium* and a non-assigned OTU (OTU 94). The comparison of the results obtained in baby faeces fermented with HT-BPL1 (both HT-BPL1 and HT-BPL1-IN) showed the highest differences. When comparing HT-BPL1-IN with the rest of the 24 h trials, a trend towards a higher percentage of the genera *Fecalibacterium, Bifidobacterium*, *Lactobacillus*, *Flintibacter* and OTU94, whereas lower percentages of *Prevotella*, *Coprococcus* and *Eubacterium* were observed. In the case of assays with 1 × 10^9^ cells HT-BPL1 per fermentation added (Figure 4B) OTU94, and genera *Fecalibacterium* and *Flintibacter* increased in fecal slurries fermented with HT-BPL1-IN while genera *Eubacterium* and *Dialister* decreased. In the case of *Coprococcus*, slurry fermentations caused an elevation of this genus, except in the case of HT-BPL1-IN, with very similar levels to those of fecal material before being fermented. On the contrary, *Akkermansia* levels dropped in all fermentations, except in the case of trials with HT-BPL1-IN assayed.

## 4. Discussion

We focused this investigation to find out whether a heat-treated choose *Bifidobacterium animalis* subsp. *lactis* CECT 8145 (BPL1) probiotic preserved some of its functional properties exhibited when alive, in a fecal model that mimics microbial fermentation in the gut. Initially, this strain showed activity lowering fat deposition both alive and heat treated in the pre-clinical *C. elegans* model [19] demonstrating the usefulness of the model for probiotic evaluation in fat deposition. After that, the activity in both formats was validated in two different pre-clinical murine models [20,21] and finally in a randomized controlled clinical trial done in adult abdominally obese subjects in which the probiotic validated previous results and showed to improve the anthropometric obesity biomarkers [27]. In the light of these positive outcomes, in this work we attempted to study the effect of strain HT-BPL1 in infant milk product.

Since the probiotic was to be incorporated into an infant milk, it was initially studied whether this strain retained its functionality in this matrix. These trials were conducted in the nematode *C. elegans*. The small size, short lifespan, complete genetic information and easy maintenance in the laboratory make *C. elegans* a valuable animal model to research. It is noteworthy that many of the genes involved in the synthesis, degradation, and transport of fats are conserved in mammals and have been identified by RNAi [28,29,30,31,32,33], being its capacity of fat accumulation dependent on the homeostasis of lipogenesis and lipolysis [34]. This nematode conserves 65% of the genes associated with human disease, and in the case of lipid and energy metabolism, many of the proteins involved in synthesis, degradation and transporting fats are highly conserved between *C. elegans* and mammals. Genetic screenings have identified many gene inactivations that cause fat reduction or fat accumulation [31]. This has led to the use of *C. elegans* used as a model to evaluate potential obesity therapeutics, and obtain mechanistic information behind single-gene mutations related to obesity, and define the mechanistic details of fat metabolism [28,29,30,31,32,33,35]. In this sense, compounds targeting conserved pathways regulating lipids in nematodes are potential successfully candidates for testing in human trials. Trials conducted in *C. elegans* model have both confirmed a dose-dependent effect in fat deposition with HT-BPL1 and that HT-BPL1 kept this property when incorporated into the infant milk product (HT-BPL1-IN). This initial result led us to further develop the model, applying added fecal slurry from the different products to analyze the evolution of the microbiome, the production of organic acids and its effect on the fat deposition model of *C. elegans* both with BPL1 alone (HT-BPL1), and with infant milk (HT-BPL1-IN), in two different cell concentrations (10^8^ cells/reactor and 10^9^ cells/reactor). In an attempt to “humanize” the intestine of *C. elegans*, nematodes have been treated with baby fermented fecal samples and results confirmed its fat-reducing effect in treated nematodes, particularly at 10^9^ cells concentration. These results suggest that microbiome impacts the physiology of the worm, influencing in health parameters. This is consistent with other *C. elegans* studies showing host-microbiome interactions, that are well stablished, like those related with oxidative stress or life-span [36]. From the metabolomic analysis, the results obtained using the fecal slurry model showed that SCFA acetate and the organic acid lactate were considerably augmented by HT-BPL1-IN, during a 24-h fermentation period. This augmentation was not observed when HT-BPL1 was added alone, suggesting that either there may be some kind of synergy between the milk powder and HT-BPL1 or most probably that the milk powder contains compounds that act on the growth and/or metabolism of different acetate and lactate producing microorganisms in the fermented infant slurries. Infant milk powder contains lactose which is can be homo-fermented or hetero-fermented by taxons such as *Bifidobacterium animalis* subsp. *lactis*, producing thus lactic and acetic acid respectively [37].

Whatever the mechanism involved, it seems that the increase in acetate has relevant impact in the context of infant obesity. SCFAs are metabolic products of microbial activity that contribute to intestinal homeostasis [38,39]. The most abundant are acetate, propionate and butyrate, that constitute more than 95% of all SCFAs [38,40]. While acetate and propionate are found in both small and large intestines, butyrate is mainly present in colon and cecum [40]. Acetate also exerts anti-inflammatory activity in *C. elegans* via NF-κB [41], also presenting functionality as immunomodulator [42]. NF-κB signaling is directly involved in the relationship of inflammation and obesity [43]. In a previous work with BPL1 in *C. elegans* model [19], it was observed that this one presented, besides a marked functionality as for reduction of the corporal fat, antioxidant and antiinflammatory activity. In the context of infant obesity, some functional observations with acetate deserve a comment. For example, the oral administration of acetate to mice fed a high-fat diet, reduced body weight, improved insulin sensitivity, decreased total body fat content and hepatic fat accumulation without changing food intake or physical activity [44]. Moreover, oral acetate gavage in an obese and diabetic strain of rats reduced weight gain and improved glucose tolerance [44,45]. Furthermore, in mice C-acetate can cross the blood-brain barrier to be taken up into the hypothalamus that resulted in decreased food intake through appetite suppression [44,45], accompanied by increased lactate production [44]. There are also some studies in humans, suggesting that acetate may affect the central regulation of appetite with concomitant reduction of body weight [44]. Concomitantly, acetate stimulates leptin secretion from adipocytes to regulate energy balance and appetite [46]. Furthermore, acetate inhibits intracellular lipolysis with reduced lipid overflow and decreased ectopic accumulation of fat [44]. This effect may reduce free fatty acid flux to the liver; in doing so, acetate decreases fatty liver risk, thus mitigating deterioration of glucose homeostasis [46].

Since SCFAs are produced as a result of the bacteria’s own metabolism of the digestive system, it is to be expected that the greatest changes at the level of bacterial populations would be observed in the fermented with HT-BPL1-IN assays. So that, microbiome study was done in order to evaluate how both HT-BPL1 and HT-BPL1-IN impact on bacterial composition. The analysis of the changes reflects a modulating effect of the microbial populations of INNOVA milk product. Although diversity varied between different samples in the control after 24 h of fermentation probably due to different initial microbial populations that evolve differentially in the fermentation process, the addition of HT-BPL1 or HT-BPL1-IN resulted in greater uniformity in diversity within each trial. Among increased groups, elevated levels of bifidobacteria and lactobacilli can enable the production of lactate and acetate. In this context, the huge increase in HT-BPL1-IN assays may be produced by the use of milk carbohydrates such us lactose in organic acids production. Some *Bifidobacterium* species preferentially use lactose over glucose as a carbon source when grown in the presence of both sugars [37,47]. Genus *Lactobacillus* was in HT-BPL1-IN fermentations although at low percentage levels, being *L. gasseri* its representative (data not shown). In the case of this species, there are studies that relate strains of *L. gasseri* with regulation of gut environment and function in a postbiotic form [48]. Finally, in both HT-BPL1 concentrations assayed, an increase in *Faecalibacterium* has been found. This genus is related to protection against inflammation and related with a healthy human gut microbiome [49], so an increase may have a protective effect against inflammatory dysbiosis and related to this, on metabolic syndrome, and thus it may be an indicator in this model of incidence in the bacterial microbiome and modulation of functionality.

The results obtained in this study show a significant effect of the product HT-BPL1-IN on fat deposition *C. elegans* model. In previous studies, preclinical obese rodents both in wistar rats fed on a cafeteria diet [20] and with genetically obese rats Zücker rats [21] confirmed the results obtained in *C. elegans* hat were later observed in humans in a clinical study, randomized double-blind study in obese patients. All in all, the current study has shown us the effect not only of the probiotic but also of the product included in its matrix, both in the microbiota and in the fat deposition of *C. elegans*, which predict, based on the previous “from worm to human” assays done, a potential effect in humans.

An ongoing clinical trial in infants fed with a milk formula supplemented with heat treated BPL1, will probably define to what extent this formula is helping to prevent the future development of obesity in those infants. An increasing number of studies have analyzed the effects of different bacterial strains on SCFA and organic acids production and infant obesity [50,51]. Our preclinical present study add evidence in favor of the potential beneficial effects of probiotics and postbiotics on the prevention of obesity, when added as supplements to milk infant formulas. 

## 5. Conclusions

In conclusion, here we show that heat-treated probiotic strain BPL1, reduces fat deposition in *C. elegans* when added to a milk infant formula as a supplement. Although there is an increase in acetate production, results here reported evidence that other mechanism underlying such effect is present. The study confirm postbiotic HT-BPL1 effect in milk formula.

## Figures and Tables

**Table 1:** Levels of short-chain organic fatty acids in slurry fermentations in the absence (control 24 h) or the presence of HT-BPL1 alone or with milk powder supplemented with HT-BPL1 (HT-BPL1-IN).

	Organic Acids Concentration (g/L)			
	Acetic Acid	Lactic Acid	Propionic Acid	Butyric Acid
Experiment 1				
Control (0 h)	2.88 ± 0.44	0.08 ± 0.11	0.87 ± 0.14	1.01 ± 0.20
Control (24 h)	3.59 ± 0.11	<LOQ ^1^	1.22 ± 0.10	1.94 ± 0.16 *
HT-BPL1 (1 × 10^8^)	3.49 ± 0.29	<LOQ ^1^	1.19 ± 0.16	1.90 ± 0.24 *
HT-BPL1-IN (1 × 10^8^)	9.89 ± 0.02 ***	8.34 ± 0.20 ***	1.11 ± 0.08	0.77 ± 0.03
Experiment 2				
Control (0 h)	7.98 ± 0.27	3.37 ± 0.21	0.78 ± 0.07	1.00 ± 0.07
Control (24 h)	8.18 ± 0.13	0.29 ± 0.03 *	1.35 ± 0.06	3.92 ± 0.03 **
HT-BPL1 (1 × 10^9^)	7.55 ± 0.34	0.10 ± 0.05 *	1.32 ± 0.28	3.78 ± 0.21 **
HT-BPL1-IN (1 × 109)	14.66 ± 1.03 **	10.62 ± 0.97 ***	0.85 ± 0.01	0.80 ± 0.03

Data are means ± SD of 2 experiments in triplicate; *** *p* < 0.001; ** *p* < 0.01; * *p* < 0.05, with respect control. ^1^ LOQ (Limit of Quantification) 0.05 g/L.

## Supplementary Materials

The following are available online at https://www.mdpi.com/2304-8158/9/5/652/s1, Figure S1: SCFAs and lactate determination by HPLC; Alpha-diversity reflected as Shannon Index; Figure S2: Alpha-diversity reflected as Shannon Index.
