# Supplementary figures and images for: An Infant Milk Formula Supplemented with Heat-Treated Probiotic Bifidobacterium animalis subsp. lactis CECT 8145, Reduces Fat Deposition in C. elegans and Augments Acetate and Lactate in a Fermented Infant Slurry
Source: Foods. 2020 May 19;9(5):652. doi: 10.3390/foods9050652 (PMC7278570; doi:10.3390/foods9050652)

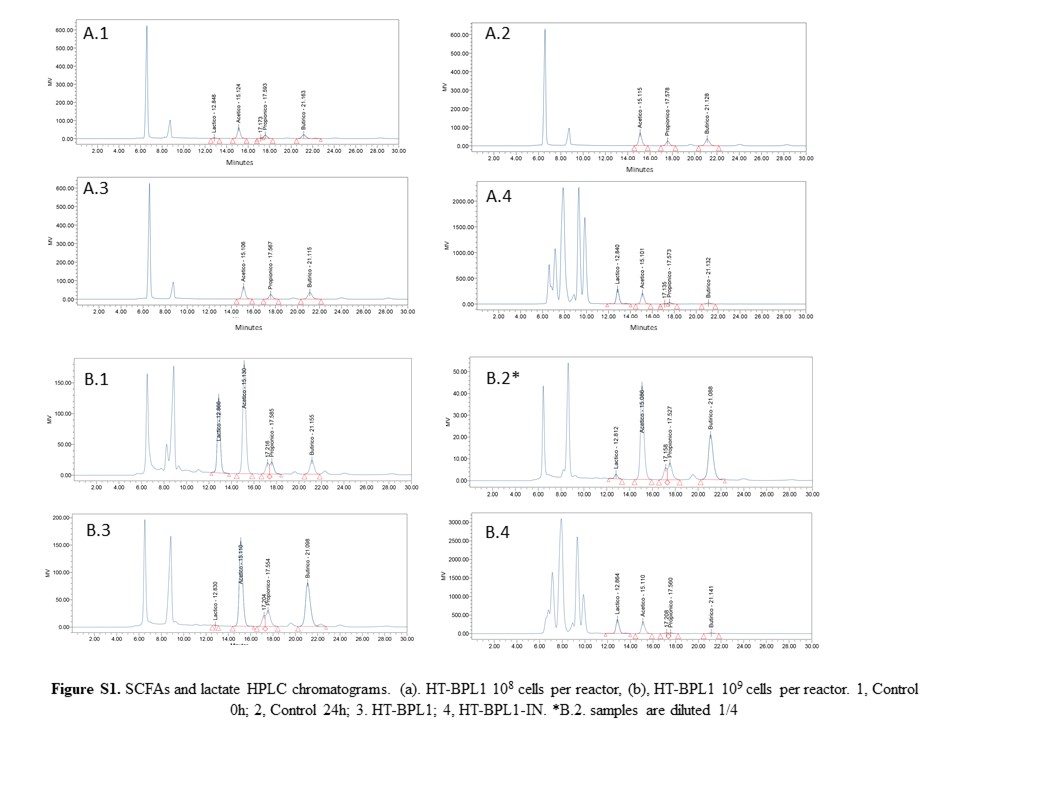

Supplement: Supplementary file 1 [file foods-09-00652-s001.zip › Figure S1.jpg]

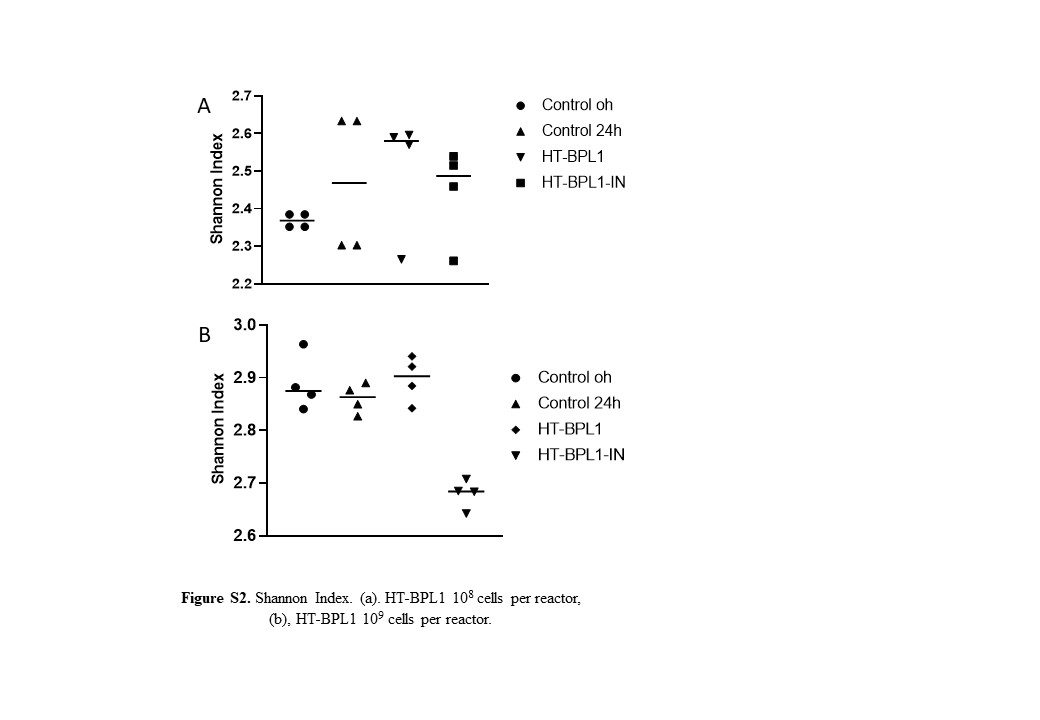

Supplement: Supplementary file 1 [file foods-09-00652-s001.zip › Figure S2.jpg]
